# Peroxiredoxin II Maintains the Mitochondrial Membrane Potential against Alcohol-Induced Apoptosis in HT22 Cells

**DOI:** 10.3390/antiox9010001

**Published:** 2019-12-18

**Authors:** Mei-Hua Jin, Jia-Bin Yu, Hu-Nan Sun, Ying-Hua Jin, Gui-Nan Shen, Cheng-Hao Jin, Yu-Dong Cui, Dong-Seok Lee, Sun-Uk Kim, Ji-Su Kim, Taeho Kwon, Ying-Hao Han

**Affiliations:** 1 College of Life Science and Technology, Heilongjiang Bayi Agricultural University, Daqing 163319, China; meihuajin7311@163.com (M.-H.J.); yjb1943@163.com (J.-B.Y.); sunhunan76@163.com (H.-N.S.); gns1980@163.com (G.-N.S.); jinchenghao3727@hanmail.net (C.-H.J.); cuiyudong1958@126.com (Y.-D.C.); 2 Library and Information Center, College of Life Science and Technology, Heilongjiang Bayi Agricultural University, Daqing 163319, China; bynd1958@163.com; 3 School of Life Sciences, KNU Creative BioResearch Group (BK21 plus project), Kyungpook National University, Daegu 41566, Korea; lee1@knu.ac.kr; 4 Futuristic Animal Resource & Research Center, Korea Research Institute of Bioscience and Biotechnology (KRIBB), Cheongju-si, Chungcheongbuk-do 28116, Korea; sunuk@kribb.re.kr; 5 Primate Resources Center, Korea Research Institute of Bioscience and Biotechnology (KRIBB), Jeongeup-si, Jeonbuk 56216, Korea

**Keywords:** alcohol, ROS, oxidative damage, Prx II, neuronal cell

## Abstract

Excessive alcohol intake can significantly reduce cognitive function and cause irreversible learning and memory disorders. The brain is particularly vulnerable to alcohol-induced ROS damage; the hippocampus is one of the most sensitive areas of the brain for alcohol neurotoxicity. In the present study, we observed significant increasing of intracellular ROS accumulations in Peroxiredoxin II (Prx II) knockdown HT22 cells, which were induced by alcohol treatments. We also found that the level of ROS in mitochondrial was also increased, resulting in a decrease in the mitochondrial membrane potential. The phosphorylation of GSK3β (Ser9) and anti-apoptotic protein Bcl2 expression levels were significantly downregulated in Prx II knockdown HT22 cells, which suggests that Prx II knockdown HT22 cells were more susceptible to alcohol-induced apoptosis. Scavenging the alcohol-induced ROS with NAC significantly decreased the intracellular ROS levels, as well as the phosphorylation level of GSK3β in Prx II knockdown HT22 cells. Moreover, NAC treatment also dramatically restored the mitochondrial membrane potential and the cellular apoptosis in Prx II knockdown HT22 cells. Our findings suggest that Prx II plays a crucial role in alcohol-induced neuronal cell apoptosis by regulating the cellular ROS levels, especially through regulating the ROS-dependent mitochondrial membrane potential. Consequently, Prx II may be a therapeutic target molecule for alcohol-induced neuronal cell death, which is closely related to ROS-dependent mitochondria dysfunction.

## 1. Introduction

Alcoholism causes multiple system damages, especially inducing the irreversible damage of brain central nervous systems, which were related to several neurodegenerative disease processes [1,2,3,4]. Alcohol has nice permeability to the blood–brain barrier, thus resulting in higher damage potentials to the central nervous system [5,6,7]. It was reported that alcohol exposure could affect the development of the central nervous system, especially of the hippocampus [8,9,10]. The hippocampus is part of the limbic system of the brain, and it is responsible for storage conversion and orientation of long-term memory. It is not only related to learning and memory, but also involved in sensory information processing, emotional control, and perceptual motor skills [11,12,13]. Previous studies have found that patients with alcoholism have a higher risk both in learning and memory disorders, as well as dementia, and the hippocampal cellular apoptosis was the most important factor in spatial memory disorders [5,14,15,16].

As the oxidation and anti-oxidation effects are unbalanced in vivo, ROS are produced excessively and exceed the body’s scavenging capacity, which damage cell membrane, organelles, and DNA, leading to cell apoptosis [17,18,19]. Alcohol can increase the sensitivity of brain to oxidative stress through selective mitochondrial damage, thereby aggravating neuronal apoptosis [20,21,22]. Cells metabolize alcohol via the CYP2E1 enzyme in neuronal cells, which is accompanied with the production of ROS, resulting in enhancing the neuronal cell cytotoxicity to alcohol treatments. It was reported that alcohol exposure can cause dysfunction of antioxidant enzymes’ expression levels, such as glutathione (GSH), superoxide dismutase (SOD), catalase, Peroxiredoxin (Prx), and so on [23], and induced the hippocampal neuronal apoptosis [20,24]. This results in irreversible damage to learning and memory [25]. Mitochondrial dysfunction, endoplasmic reticulum stress (ER stress), autophagy, and death receptor pathways can induce cell apoptosis, and most studies have shown that alcohol-induced apoptosis belongs to the mitochondrial pathway [26]. When cells are attacked by alcohol, ROS is produced by alcohol’s metabolite and acetaldehyde, and promote the mitochondria-dependent cellular apoptosis [27].

Prxs is peroxide reductase widely distributed in various cell types. It has functions for removing the low level of ROS, and plays important role in cell proliferation and apoptosis [28,29,30]. Our previous studies have shown that the Prxs family have different locations and expression levels in the central nervous system, and the Prx II is expressed abundantly in neurons [31]. We also reported that Prx II is essential for maintaining synaptic plasticity and exhibits a protective role in neuronal cells against aging through scavenging the cellular ROS [32]. However, the possible regulatory role of Prx II in alcohol-induced hippocampal neuronal cells apoptosis has yet not been clarified. In this study, we investigated the regulatory function of Prx II on alcohol-induced apoptosis and mitochondria dysfunction in HT22 cells. Furthermore, to understand the possible mechanisms of Prx II on alcohol-induced HT22 cell apoptosis, we also checked the ROS/GSK signaling pathways, which were correlated with cellular apoptosis and mitochondria functions.

## 2. Materials and Methods 

### 2.1. Chemicals and Reagents

Methyl thiazolyltetrazolium (MTT) and conventional reagents of cell culture were procured from Gibco (Thermo Fisher Scientific, Waltham, MA, USA). Cell Counting Kit-8 was procured from MCE (Shanghai, China). Antibodies for Western blotting were procured from Cell Signal Technology (Danvers, MA, USA). AnnexinV and DHE-DCFDA was purchased from Beyotime Biotechnology (Shanghai, China). Hoechst and MitoSOX were procured from Invitrogen (Thermo Fisher Scientific, Waltham, MA, USA). Other chemicals and reagents were purchased from local suppliers and were of analytical grade.

### 2.2. Cell Culture 

The immortalized mouse hippocampal cell line, HT-22 Mouse Hippocampal Neuronal Cell Line (HT22), was cultured in standard DMEM that was supplemented with 10% FBS, 1% penicillin/streptomycin at 37 °C under a 95% air/5% CO_2_ incubator. Culture medium was changed every day when it reached 90% confluence, and cells were sub-cultured after treatment with 0.25% trypsin–EDTA mixture. 

### 2.3. Transduction of HT22 Cells with Lentiviral-Delivered Prx II Wild Type and Prx II siRNA 

Constructing Lentiviral vectors to rescue (for Prx II siRNA) and knockdown of mouse Prx II gene, the Lentiviral constructs expressing Prx II siRNA were purchased from Suzhou Genepharma Co., Ltd., China. The sequence for over-expressing of mouse Prx II is as follows: 5’- GGATGGTGCCTTCAAGGAAAT -3’ (wtPrx II), and the sequence for knockdown of mouse Prx II siRNA is as follows: 5’- GCAAGGAATACTTCTCCAAAC -3’ (shPrx II) [33]. HT22 cells grown to 80% confluence in six-well plates, and then Prx II-siRNA-lentivirus were transduced at multiplicity of infection (MOI) of 60 using polybrene (10 µg/mL) (Genepharma, Shanghai, China). The negative control virus lentivirus (Genepharma, Shanghai, China) was transduced into HT22 cells using the same methods. Knockdown level of Prx II was detected with Western blot.

### 2.4. Cell Viability Assay

The HT22 cells were cultured in 96-well plates at a density of 5 × 10^3^ cells per well, and incubated for 24 h. Cell viability was determined with 3-(4,5-dimethyldiazol-2-yl)-2,5-diphenyltetrazolium bromide (MTT) assay, and Cell Counting Kit-8 (CCK-8). For alcohol treatment, cells were cultured in medium containing different concentrations of alcohol (0–600 mM). After treatment durations, 0.5mg/mL of the MTT or 10 μL of the CCK-8 were added to each well and incubated for 4 h at 37 °C in 5% CO_2_, and then the absorbance was determined at 540 nm or 450 nm using a microplate reader (AOE Instruments, Shanghai, China). All control and alcohol-treated plates were wrapped with parafilm to prevent evaporation of alcohol.

### 2.5. Detection of ROS and Superoxide Anion 

The intracellular ROS and superoxide anion were measured by DHE and MitoSOX probes in live cells, respectively. Briefly, treated cells cultured were washed and incubated with DHE or Hoechst/MitoSOX for 30 min at 37 °C. After reaction, cells were washed and observed by flow cytometry (Backman Coulter Commercial Enterprise, Shanghai, China) or fluorescence microscopy for detecting the ROS levels in intracellular and superoxide anion in mitochondria. All data and images were obtained from three independent trials.

### 2.6. Flow Cytometric Detection of Mitochondrial Membrane Potential

The fluorescent probe JC-1 was used to measure mitochondrial membrane potential of HT22. In terms of the functioning of the assay, the JC-1 probe accumulates in the mitochondrial matrix to form a polymer, which results in marked red fluorescence in normal mitochondria; however, the JC-1 probe exists as a monomer in damaged mitochondria, and appears as marked green fluorescence. The HT22 cells were cultured in a six-well culture plate (same amount of cells per well) with DMEM supplemented with 10% FBS. Cells were exposed to alcohol (200 mM, 400 mM) for 1 h to measure the mitochondrial membrane potential. The HT22 cells were washed with PBS; 300 µL of JC-1 staining solution (Abcam, Cambridge, UK) was added to each well; and cells were incubated for 15 min at 37 °C, protected from light. The cells were washed twice with PBS and analyzed by flow cytometry and fluorescence photography (Leica Microsystems, Wetzlar, Germany).

### 2.7. Detection of Apoptosis 

The HT22 cells were cultured in six-well plates at the concentration of 2 × 10^5^/well, and incubated for 24 h. Cells were exposed to alcohol (200 mM, 400 mM) for 1 h to measure the apoptosis level. The cells were washed with PBS and then incubated with Annxin V-PE (500 μL cell binding buffer + 1 μL Annxin V-PE) for 15 min. The cells were washed twice with PBS and analyzed by flow cytometry and fluorescent photography.

### 2.8. Western Blot 

Total protein from HT22 cell lysates was subjected to sodium dodecyl sulfate polyacrylamide gel electrophoresis using 12% SDS–polyacrylamide gel, and was then electrophoretically transferred to nitrocellulose membranes. Membranes were blocked in 5% skim milk in TBST and then incubated overnight at 4 °C with anti-Prx II, anti-Prx I anti-Bad, anti-Bax, anti-Bcl2, anti-cleaved-caspase 3, anti-pro-caspase 3, anti-β-actin, anti-pGSK3β(Ser9), anti-GSK3β, and anti-β-catenin, which were all purchased from Santa Cruz (Santa Cruz Biotechnology, Dallas, TX, USA). Membranes were washed and incubated with HRP-conjugated secondary antibody for 2 h at room temperature. After removal of excess antibodies by washing with TBST, specific binding was detected using a chemiluminescence detection system (General Electric Company, Shanghai, China). Band intensities were quantified using the Image J software (National Institutes of Health, Bethesda, MD, USA).

### 2.9. Electron Microscopy Analysis 

The HT22 cells were exposed to alcohol, and immersed in a fixative made up of 4% paraformaldehyde and 1% glutaraldehyde at 4 °C overnight. The cells were then rinsed in the PBS buffer and postfixed in 1% osmium tetroxide. Postfixation was followed by dehydration in ethanol, embedding in Epon, and polymerization. The cells were cut using an ultramicrotome, stained with 1% uranyl acetate and lead citrate, and examined under a transmission electron microscope (JEOL JEM-2100Plus, Nippon electronics, Tokyo, Japan).

### 2.10. Statistical Analysis

Repeated measures analysis of variance (ANOVA) was used to analyze changes across time and differences between groups in each experiment. Data for the experiment were measured using independent-samples *t*-test. All differences were considered statistically significant if the *p*-value was less than 0.05 (* *p* < 0.05; ** *p* < 0.01; *** *p* < 0.001).

## 3. Results

### 3.1. Prx II Knockdown Elevated Alcohol Induced Apoptosis and ROS Accumulation in HT22 Cells

**Figure 1:**
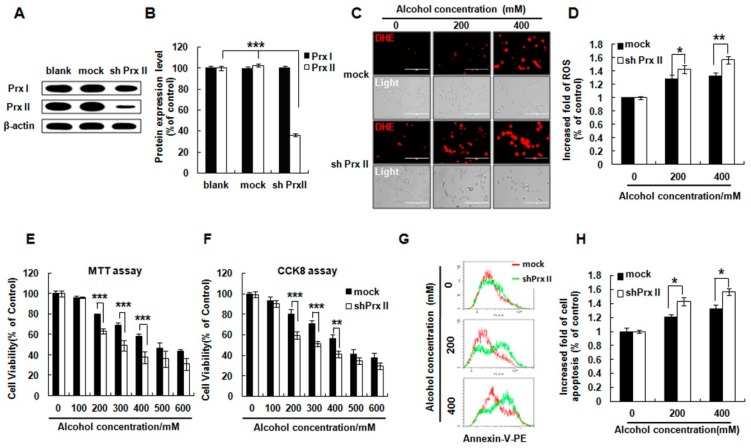
Effect of Peroxiredoxin II (Prx II) on cell viability, cellular ROS, and apoptosis in HT22 cells after alcohol stimulation. (**A**) The Western blot analysis of Prx I and Prx II expression in blank, mock, and shPrx II HT22 cells. (**B**) The protein expression levels were quantified using ImageJ software and the differences are represented by histogram; β-actin was used as a loading control (means ± SE of three independent experiments) (** *p* < 0.01) (**C**) The cellular ROS levels were detected by DHE (red) staining, a dye for cellular ROS detection (Scale bar = 200 μm). (**D**) Intracellular ROS production was measured by flow cytometry following staining with DHE dye; bar graphs show quantitative analysis of mean values from three independent experiments. (* *p* < 0.05; ** *p* < 0.01). (**E**,**F**) Mock HT22 cells and shPrx II HT22 cells were cultured in different concentrations of alcohol (0–600 mΜ) for 24 h. Cell viability was measured by 3-(4,5-dimethyldiazol-2-yl)-2, 5-diphenyltetrazolium bromide (MTT) and Cell Counting Kit-8 (CCK8) assay. Each value represents the mean (±SEM) from at least three independent experiments (*** *p* < 0.001, ** *p* < 0.01). (**G**,**H**) Cellular apoptosis was measured with flow cytometry as fluorescence intensity of Annexin V-PE. The bar graphs show the quantitative analysis of mean values in (**G**) from three independent experiments (* *p* < 0.05).

Prx I and Prx II protein expression levels were detected in HT22 cells. As shown in Figure 1A and B, the Prx II protein expression was significantly decreased in Prx II shRNA transfected HT22 cells, while there were no changes in Prx I protein expression. It was reported that alcohol can produce excessive ROS in neuronal cells through an enzyme system or a non-enzymatic system [21]. As shown in Figure 1C,D, the levels of ROS in both groups were significantly increased by the alcohol treatment with indicated concentrations (0, 200, 400 mM) and severe ROS accumulations were observed in Prx II knockdown cells compared with that of the mock cells. Cell viability was detected by MTT assay and CCK8 kit. The results showed that knockdown of Prx II significantly decreased the cell viability upon to alcohol treatment compared with mock cells (Figure 1E,F). To verify whether Prx II knockdown could influence the alcohol induced cell apoptosis, the mock and shPrx II cells were treated with alcohol (0, 200, and 400 mM), and the cellular apoptosis was analyzed with flow cytometry by detecting the Annexin-V fluorescence. The result shows that alcohol treatment significantly increased the cellular apoptosis in both types of HT22 cells, and Prx II knockdown led to enhancing the apoptosis compared with mock cells (Figure 1G,H). These results indicate the protective role of Prx II on alcohol-induced cell death in HT22 cells.

### 3.2. Knockdown of Prx II Elevated the Alcohol-Induced Mitochondria ROS and Membrane Permeability in HT22 Cells 

**Figure 2:**
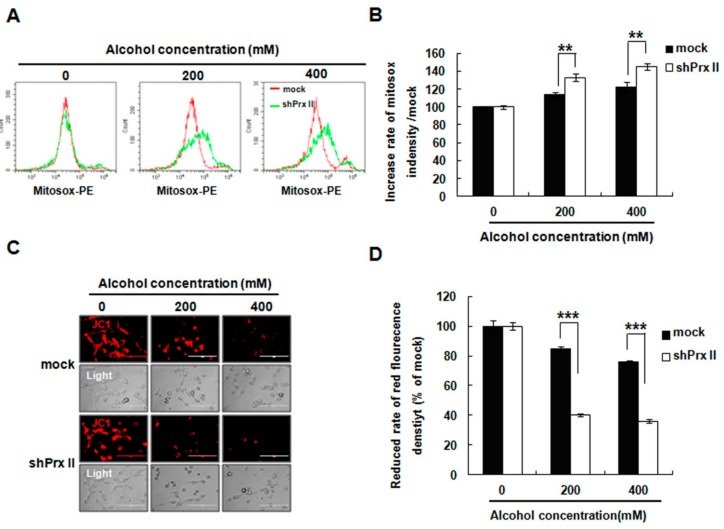
Effect of Prx II on mitochondrial ROS and membrane permeability in HT22 cells after alcohol stimulation. (**A**,**B**) The mitochondria superoxide anion was measured by flow cytometry following stinging MitoSOX and the quantified data are shown as a graph in (**B**) (** *p* < 0.01). (**C**) The mitochondrial membrane potential was measured by JC-1 staining and observed with fluorescence microscopy. (**D**) The fluorescence intensity was measured by flow cytometry following staining with JC-1 dye, and bar graphs show quantitative analysis of mean values from three independent experiments. (*** *p* < 0.001).

The reports indicate that mitochondria are susceptible to ROS exposure, and the oxidative stress causes mitochondrial permeability transition pores (MPTPs) to open, and increased the mitochondrial membrane permeability, leading to loss of key functions and induction of apoptosis. MitoSOX (a dye for mitochondrial ROS detection) staining results indicated that mitochondria ROS accumulation was more abundant in the Prx II knockdown cells compared with the mock cell (Figure 2A,B). To determine whether the mitochondrial permeability transition (MPT) was caused by alcohol treatment, MPT was conducted by JC-1 staining. As shown in Figure 2C,D, JC-1 fluorescence was significantly decreased in Prx II knockdown HT22 cells compared with mock cells after alcohol treatment. 

### 3.3. Knockdown of Prx II Results in Up-Regulating the Mitochondria Dependent Cellular Apoptosis

**Figure 3:**
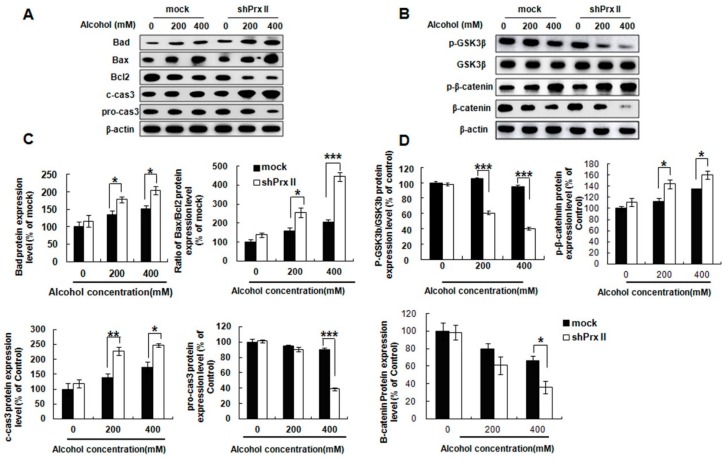
Effect of Prx II on apoptosis relate proteins expression. (**A**,**C**) Protein expression level of Bad, Bax, Bcl2, c-cas3, and pro-cas3 were observed by Western blotting analysis in mock and shPrx II HT22 cells after alcohol treatment. (**B**,**D**) Western blotting analysis for p-GSK3β, GSK3β, p-β-catenin, and β-catenin in mock and shPrx II HT22 cells after alcohol treatment. Each value represents the mean (±SEM) from at least three independent experiments. (* *p* < 0.05; ** *p* < 0.01; *** *p* < 0.001).

To understand the possible molecular mechanism of the alcohol induced HT22 cell apoptosis in Prx II knockdown cells, we also examined the expression levels of apoptosis-related proteins. The results showed that pro-apoptotic proteins Bad, Bax, and cleaved caspase 3 expressions were significantly increased in the Prx II knockdown HT22 cells compared with mock cells, while the anti-apoptotic protein Bcl2 and pro-caspase 3 expression levels were significantly decreased (Figure 3A,C). It was reported that GSK3β is a crucial positive regulator of the MPTP and also a principle trigger of cell death [34]. Thus, the GSK/β-catenin signal pathway was also examined in mock and Prx II knockdown HT22 cells after alcohol treatment. The result shows (Figure 3B,D) that phosphorylated GSK3β (Ser9) and β-catenin were significantly decreased after alcohol treatment in shPrx II HT22 cells, while the phosphorylated β-catenin were significantly increased compared with mock HT22 cells. 

### 3.4. Regulatory Function of Prx II on Alcohol-Induced Mitochondria Dysfunctions is Dependent on Cellular ROS Levels

**Figure 4:**
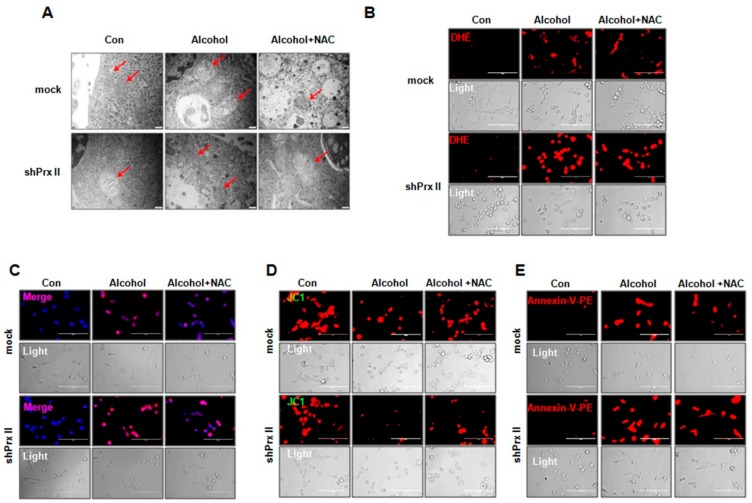
Regulatory function of Prx II on alcohol-induced mitochondria dysfunctions is dependent on cellular ROS levels (**A**) Mitochondrial morphology was observed by transmission electron microscopy (magnification 20,000×). Mitochondria are indicated with red arrowheads. (**B**) Intracellular ROS production was measured by fluorescence microscope stained with DHE (red), scale bar = 200 μm. (**C**) The mitochondria superoxide anion was measured by fluorescence microscope following stinging Hoechst/MitoSOX (blue/red), scale bar = 200 μm. (**D**) Mitochondrial membrane potential was measured with fluorescence microscopy (JC-1 staining). (**E**) Cellular apoptosis was measured by fluorescence microscopy stained with Annexin V-PE (red), scale bar = 200 μm.

As alcohol treatment could significantly increase the cellular and mitochondria ROS levels, which led to mitochondrial damage and cell apoptosis, we hypothesize that scavenging the ROS should influence the alcohol induced cellular apoptosis. To verify this, the shPrx II HT22 cells were pre-treated with NAC, a ROS scavenger, for 30 min flowing treated with alcohol for 1 h, and the mitochondrial morphological changes were observed by transmission electron microscopy. As shown in Figure 4A, mitochondria are enlarged and swollen, and cristae vague appear in the Prx II knockdown HT22 cells compared with mock cells after alcohol treatment and reversed by NAC treatment. Intracellular ROS levels were significantly decreased after NAC treatment both in alcohol-treated mock and Prx II knockdown HT22 cells (Figure 4B), and the mitochondria ROS levels were also eliminated (Figure 4C). In addition, NAC treatments significantly reversed the alcohol-induced increase of the mitochondria ROS levels (Figure 4D). Fortunately, scavenging the ROS with NAC also significantly restored the alcohol-induced apoptosis in Prx II knockdown HT22 cells (Figure 4D). These results indicate that Prx II protects the mitochondria by eliminating intracellular ROS induced by alcohol.

### 3.5. NAC Treatment Restores the Alcohol-Induced Up-Regulating of the Apoptosis-Related Protein Expressions in shPrx II HT22 Cells

**Figure 5:**
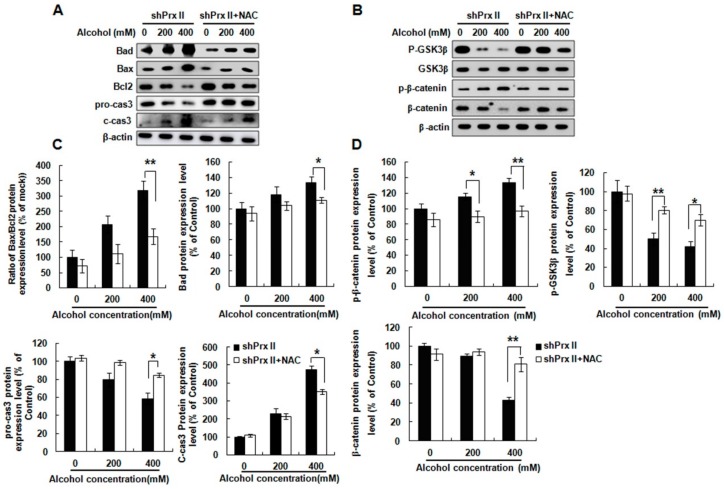
Effect of NAC treatment on apoptosis-related protein expression in Prx II knockdown HT22 cells after alcohol treatment. (**A**,**C**) The apoptosis related protein expressions were analysed with Western blot. (**B**,**D**) Western blotting analysis for p-GSK3β, GSK3β, p-β-catenin, and β-catenin protein expressions in shPrx II cells after alcohol treatment. All the results were done at least three times with independent experiments (* *p* < 0.05; ** *p* < 0.01).

We also examined the effect of NAC treatment on apoptosis-related proteins’ expression levels, such as Bad, Bax, Bcl2, pro-caspase 3, and cleaved-caspase 3 in Prx II knockdown HT22 cells. As shown in Figure 5A,C, treatment of NAC significantly reversed the alcohol-induced increase of Bad, Bax, and cleaved-caspase 3 protein expressions, and up-regulated Bcl2 and pro-caspase 3 protein expressions. Fortunately, the expressions of phosphorylated GSK3β (Ser9) and β-catenin levels were also recovered; the phosphorylated β-catenin levels were suppressed (Figure 5B,D). These results suggest that clearance of the intracellular ROS can prevent apoptosis of Prx II knockdown HT22 cells induced by alcohol stimulation.

### 3.6. Prx II Inhibits the Cellular Apoptosis by Down-Regulating the Mitochondria-Dependent Apoptotic Pathway

**Figure 6:**
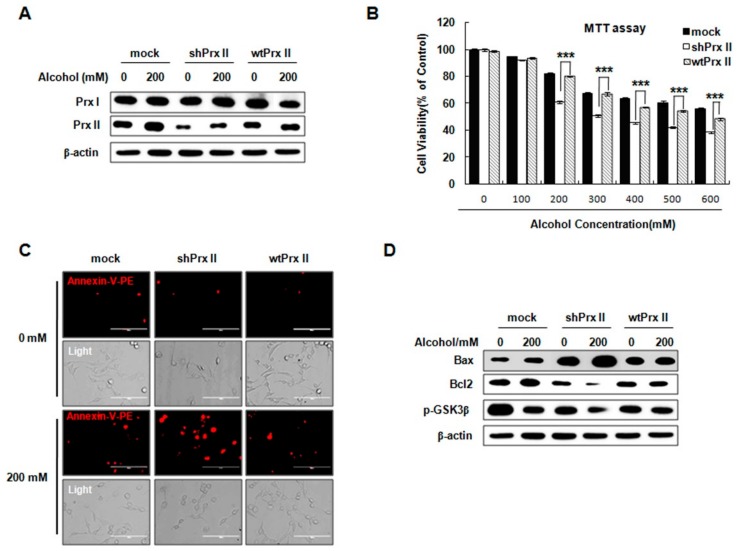
Prx II inhibit the cellular apoptosis by downregulating the mitochondria-dependent apoptotic pathway. (**A**) The Western blot analysis of Prx I and Prx II expression in mock, shPrx II, and wtPrx II HT22 cells. (**B**) Mock HT22 cells, shPrx II HT22 cells, and wtPrx II HT22 cells were cultured in different concentrations of alcohol (0–600 mΜ) for 24 h. Cell viability was measured by MTT. Each value represents the mean (± SEM) from at least three independent experiments (***p < 0.001). (**C**) Cellular apoptosis was measured by fluorescence microscopy stained with Annexin V-PE (red), scale bar = 200 μm. (**D**) Western blotting analysis of Bax, Bcl2, and p-GSK3β.

This study showed that the NAC-mediated scavenging of intracellular ROS significantly inhibited cellular apoptosis in Prx II knockdown HT22 cells exposed to alcohol. In order to verify the role of Prx II, we have re-expressed Prx II in Prx II knockdown HT22 cells (Figure 6A). As shown in Figure 6A, the Prx II protein expression level was significantly increased in wtPrx II HT22 cells compared with shPrx II HT22 cells, but there was no significant change in the Prx I protein expression level. The cell viability analysis showed that rescuing the Prx II on shPrx II cells significantly restores the decrease of cell viability response to alcohol treatment (Figure 6B). In addition, re-expressed Prx II also significantly restored the alcohol induced apoptosis in Prx II knockdown HT22 cells (Figure 6C), significantly reversed the alcohol-induced increase of Bax protein expressions, and upregulated Bcl2 and phosphorylated GSK3β (Ser9) levels (Figure 6D). These results suggest that Prx II protects cells from alcohol stimulated apoptosis in HT22 cells.

## 4. Discussion

The present study showed that Prx II knockdown HT22 cells were sensitive to acute alcohol induced cell damage, as evidenced by increased intracellular levels of ROS, activated GSK-3β/β-catenin signaling pathway, and decreased mitochondrial membrane potential, which eventually lead to cell apoptosis. Our finding demonstrated that Prx II plays a crucial role in maintaining the mitochondrial membrane potential by alcohol-mediated ROS, and protecting hippocampal neuron cells against alcohol-induced cell apoptosis.

In the past few decades, researchers have conducted in-depth studies on the causes and related mechanisms of alcohol-induced neuronal cell death in several aspects [9,12,35,36]. Many studies on experimental animal models provide evidence that the brain is susceptible to alcohol toxicity, especially in the cerebral cortex and hippocampus [37,38,39,40,41]. Alcohol enters the nervous system and increases intracellular ROS, leading to apoptosis of hippocampal neurons, which in turn leads to cognitive and memory impairment and neurodegenerative diseases, such as alcoholic-associated dementia [42,43,44,45]. Earlier studies have shown that the main cause of alcoholic learning and memory impairments is damage to the hippocampus by ROS [43,46,47]. Therefore, exploring the mechanism of learning and memory impairment induced by apoptosis of hippocampal neurons with alcohol, and understanding the relationship among ROS, mitochondrial, antioxidant system, and neuronal cell apoptosis, can provide potential therapeutic targets for alcoholic brain damage. According to the legally intoxicating level of alcohol (approximately 22 mM) in the United States, the levels of alcohol used in cellular systems in general are four to five times higher than the legally intoxicating level; this study focused on neurological damage caused by acute alcohol intoxication, so higher concentrations of alcohol were used [24,48].

Our study showed that alcohol treatment resulted in a significant increase of intracellular and mitochondrial ROS levels in HT22 cells in a concentration-dependent manner, and that NAC treatment restored the alcohol induced upregulating of the apoptosis-related protein expressions in shPrx II HT22 cells. These results indicate that ROS accumulation is the main cause of alcohol-induced apoptosis in Prx II knockdown HT22 cells. It is well established that the Prx family of enzymes, glutathione peroxidase, and catalase play an important role in neutralization of intracellular ROS. The Prx family has a lower catalytic efficiency (about 10^5^ M/S) and high affinity for ROS (about 10^8^ M/S) relative to glutathione peroxidase (about 10^8^ M/S) and catalase (about 10^6^ M/S) [49,50]. Prx I and Prx II are highly homologous 2-cysteine members of the Prxs family that function as antioxidants at low resting H_2_O_2_ levels. Nevertheless, knockdown of Prx II did not affect the expression of Prx I in HT22 cells in our experiments, suggesting the increased ROS, apoptosis, and mitochondrial ROS levels were mainly affected by the Prx II knockdown after alcohol treatment. Furthermore, our previous research has reported that knockout of Prx II causes age-dependent mitochondrial ROS accumulations in mice, which results in failing to activate the synaptic plasticity of the neurons through CREB, CaMKII, and ERK signaling pathways [29]. In the present study, we used the alcohol as a stimulator for inducing the apoptosis of HT22 cells, which accompanied the acute increasing mitochondrial ROS accumulations and decrease in the mitochondrial potentials by alcohol treatments, and ultimately led to cellular apoptosis. Moreover, the phosphorylation of GSK3β (Ser9) was significantly decreased in Prx II knockdown HT22 cells after alcohol stimulation. GSK3β is a multifunctional serine kinase that is involved in a variety of pathobiological functions and has also been reported in neuronal damage [51,52]. Activated GSK3β induces phosphorylation of VDAC1, promotes the opening of MPTP, reduces mitochondrial membrane potential, increases cytochrome C release, and ultimately induces cell apoptosis [52,53,54]. According these findings, we can make a conclusion that Prx II plays a critical role in protecting the mitochondria from oxidative stress in neuron cells, and GSK3β is the key molecule in ROS-mediated mitochondria dysfunction.

**Figure 7:**
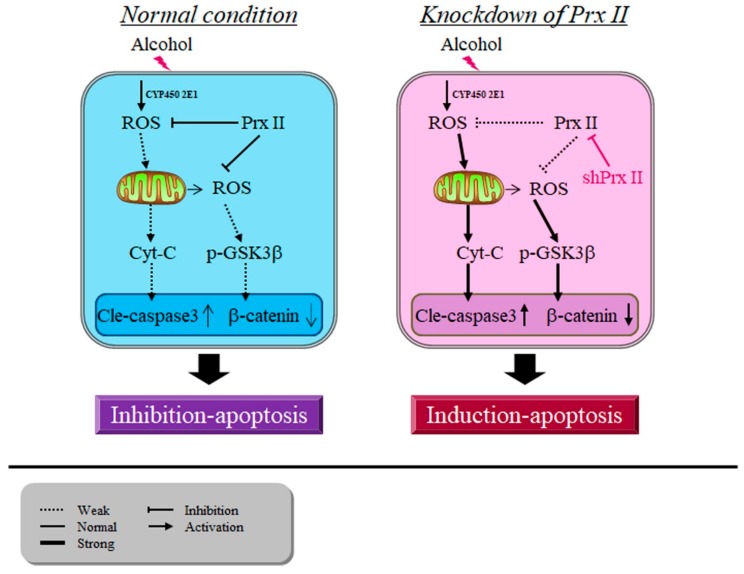
Peroxiredoxin II maintains the mitochondrial membrane potential against alcohol-induced apoptosis.

Removing intracellular ROS from alcohol-treated Prx II knockdown HT22 cells by NAC can significantly prevent mitochondrial membrane potential decrease and GSK3β dephosphorylation. Prx II could scavenge the alcohol-induced ROS accumulations, which were produced by both CYP450 or mitochondria, and protect the neuronal cells from the alcohol toxicity partially. However, in the event of knockdown Prx II, the alcohol-mediated ROS could severely accumulate in the cells, which results in the activation of both the mitochondria and GSK signaling mediated apoptosis in HT22 neuronal cells, and all of these phenomena may be thanks to the dysfunction of mitochondria, stimulated by alcohol treatments (Figure 7). GSK3β and apoptosis involving the mitochondria-mediated intrinsic apoptotic signaling cascade can be induced by numerous stimuli that cause cell damage, such as oxidative stress, ER stress, and DNA damage, among others [55,56]. From the relationship between ROS and mitochondrial membrane potential changes in this study, the increase of alcohol-induced ROS in Prx II knockdown cells is the main cause of cell apoptosis. The activation of GSK3-β was also regulated by the PI3k/AKT signaling pathway [54]. Thus, we also have a suspicion that, once Prx is knocked down, it directly inhibits the PI3K/Akt signaling pathway, GSK-3β dephosphorylation causes a change in mitochondrial membrane potential that leads to HT22 cell apoptosis, but this requires further study to clarify.

## 5. Conclusions

In conclusion, Prx II knockdown HT22 cells were sensitive to alcohol-induced apoptosis, and alcohol-induced intracellular ROS activate the GSK-3β/β-catenin signaling pathway and decrease the mitochondrial membrane potential; all of these findings suggest that Prx II maintains mitochondrial membrane potential by eliminating alcohol-induced ROS and protects HT22 cells from alcohol-induced damage. However, the intrinsic detailed mechanisms of the regulatory role of Prx II in the GSK-3β/β-catenin signaling pathway need to be investigated.
